# Hyaluronic Acid and a Short Peptide Improve the Performance of a PCL Electrospun Fibrous Scaffold Designed for Bone Tissue Engineering Applications

**DOI:** 10.3390/ijms22052425

**Published:** 2021-03-12

**Authors:** Dana Rachmiel, Inbar Anconina, Safra Rudnick-Glick, Michal Halperin-Sternfeld, Lihi Adler-Abramovich, Amit Sitt

**Affiliations:** 1 Department of Oral Biology, The Goldschleger School of Dental Medicine, Sackler Faculty of Medicine, Tel Aviv University, Tel Aviv 6997801, Israel; danarach22@gmail.com (D.R.); safrar@gmail.com (S.R.-G.); michal4@mail.tau.ac.il (M.H.-S.); 2 The Center for Nanoscience and Nanotechnology, Tel Aviv University, Tel Aviv 6997801, Israel; inbar.anco@gmail.com; 3 Department of Physical Chemistry, The School of Chemistry, Raymond and Beverly Sackler Faculty of Exact Sciences, Tel-Aviv University, Tel Aviv 6997801, Israel

**Keywords:** bone tissue engineering, electrospinning, self-assembly, short peptide, hyaluronic acid, scaffolds

## Abstract

Bone tissue engineering is a rapidly developing, minimally invasive technique for regenerating lost bone with the aid of biomaterial scaffolds that mimic the structure and function of the extracellular matrix (ECM). Recently, scaffolds made of electrospun fibers have aroused interest due to their similarity to the ECM, and high porosity. Hyaluronic acid (HA) is an abundant component of the ECM and an attractive material for use in regenerative medicine; however, its processability by electrospinning is poor, and it must be used in combination with another polymer. Here, we used electrospinning to fabricate a composite scaffold with a core/shell morphology composed of polycaprolactone (PCL) polymer and HA and incorporating a short self-assembling peptide. The peptide includes the arginine-glycine-aspartic acid (RGD) motif and supports cellular attachment based on molecular recognition. Electron microscopy imaging demonstrated that the fibrous network of the scaffold resembles the ECM structure. In vitro biocompatibility assays revealed that MC3T3-E1 preosteoblasts adhered well to the scaffold and proliferated, with significant osteogenic differentiation and calcium mineralization. Our work emphasizes the potential of this multi-component approach by which electrospinning, molecular self-assembly, and molecular recognition motifs are combined, to generate a leading candidate to serve as a scaffold for bone tissue engineering.

## 1. Introduction

Bone is a metabolically active connective tissue capable of self-regeneration through the process of bone remodeling. Bone defects may be the result of trauma, neoplasms, congenital defects, or bone-associated diseases. When the damage is great, large defects cannot self-repair and, thus, therapeutic intervention is required [1]. In such cases, autologous bone graft is regarded as the gold-standard of treatment; however, this method has certain drawbacks, including secondary damage, high donor site morbidity, and limited availability. Similarly, other intervention options, such as allografts and xenografts, may be associated with immune-mediated rejection and transmission of diseases [1,2,3]. Bone tissue engineering is an alternative approach in which the damaged bone is regenerated with the aid of a biomaterial scaffold. The objective of tissue engineering is to construct a bio-artificial scaffold that mimics the ECM structure and function, and therefore supports cellular adhesion, proliferation, and differentiation, in addition to providing the required physical properties [1,2,3,4]. Scaffolds that demonstrate high porosity, interconnected pores, and a large surface area, together with sufficient mechanical and morphological properties to make them load-bearing, are considered superior [4,5,6].

In the last two decades, electrospinning has emerged as a leading approach for the formation of scaffolds suitable for the growth of different tissues. This is mainly because the polymeric nanofibrous nonwoven meshes produced by electrospinning possess high porosity and exhibit mechanical and functional properties that resemble those of the ECM [4,6]. Electrospun scaffolds can be prepared from a variety of polymers, including biopolymers and synthetic biodegradable and biocompatible polymers, offering a wide range of mechanical, chemical, and biological properties that can be tailored to the growth of the tissue [4,7,8,9]. Electrospinning also allows different polymers to be combined in the same fiber, thereby producing fibers with versatile architectures, including core/shell and pie-like structures [10]. Furthermore, electrospinning enables the incorporation of active molecules, including drugs and growth factors, which can further improve the compatibility and performance of the scaffold.

Polycaprolactone (PCL) is a synthetic biodegradable polyester that is widely used in tissue engineering because of its availability, relatively low price, and its ability to be chemically modified [11,12]. The polymer’s elastic properties and ease of use in composites make it an excellent candidate for electrospinning fibers. The degradation of PCL is relatively slow; complete degradation of the ester bonds by lipase enzymes could take up to two to three years in biological systems. This long half-life makes it a good candidate for tissue engineering of hard tissues, where healing requires an extended period of time [13,14]. However, synthetic polymers lack many of the properties required for tissue engineering and specifically, the hydrophobic surfaces and the absence of cellular recognition sites fail to promote cellular attachment [6,13,15]. This issue may be addressed by the recent introduction of polymer–polysaccharide composite scaffolds [14,16,17].

Hyaluronic acid (HA) is a large polysaccharide composed of repeating disaccharides units. HA is present in large quantities in the ECM and plays a significant role in many biological processes, including cell proliferation, molecular signaling, and wound healing [18,19]. The material has been extensively studied in the field of tissue engineering, since it has been demonstrated that incorporating HA improves the cellular migration and proliferation in the scaffolds [16]. However, despite the excellent biocompatibility, HA has relatively low mechanical strength, and as a polyelectrolyte also has a significant amount of free charge carriers. These properties make electrospinning pure HA highly challenging, and thus HA is usually combined with another polymer, e.g., PCL, to prepare an electrospun scaffold [14,18].

Scaffolds made of HA can also be strengthened by utilizing molecular self-assembly [18,20,21]. Many biomaterials spontaneously self-assemble through noncovalent interactions to produce well-ordered structures [20,22,23,24,25,26]. Short peptides can form three-dimensional (3D) hydrogels that support cellular attachment and proliferation. One of these low-molecular-weight hydrogelators, fluorenyl-methoxycarbonyl-diphenylalanine (FmocFF) peptide, which has been studied extensively [2,18,23,24,25,27,28,29], assembles efficiently into an ordered tubular structure and forms a rigid fibrous hydrogel under physiological conditions [2,18,25]. Combining self-assembled short peptides with polysaccharides has been reported to improve the mechanical properties of 3D hydrogels [18,20,21,22,23]. A recent study reported that co-assembly of HA polymer with the supramolecular hydrogelator FmocFF could produce a biocompatible hydrogel with excellent biological properties and superior mechanical properties emanating from the FmocFF groups [18].

Another low-molecular-weight hydrogelator, Fmoc-phenylalanine-arginine-glycine-aspartic acid (FmocFRGD), self-assembles to form a fibrous network [27,28]. The RGD sequence in FmocFRGD is a motif present in a variety of extracellular matrix proteins and serves as a molecular recognition sequence that can stimulate the attachment and spreading of cells like fibroblasts and endothelial cells on various coated substrates [15,30,31]. Moreover, specifically in the field of bone tissue engineering, incorporating the RGD molecular recognition sequence in bone implants results in an increase in bone growth, a decrease in the development of interface fibrous tissue, faster cellular differentiation into osteoblasts, and a decrease in the time required for osseointegration. Notably, better results were obtained when RGD was incorporated with natural polymers, such as HA [32]. The interaction between integrins and ECM proteins can be mimicked by using short synthetic peptides containing the RGD sequence. Hence, in recent years, efforts have focused on incorporating the RGD sequence into peptide modified biomaterial [28].

Here, we describe a fibrous scaffold fabricated via coaxial electrospinning PCL polymer together with HA, and incorporating the FmocFRGD peptide. We characterized the electrospun nonwoven meshes of fibers, using electron microscopy, and demonstrated the ability of the scaffold to mimic the fibrillary structure of the ECM. The addition of HA and FmocFRGD to the scaffold improved the bioactivity and cell affinity, and facilitated osteogenic differentiation, as demonstrated by intracellular Alkaline Phosphatase (ALP) activity and biomineralization.

## 2. Results and Discussion

### 2.1. Preparation and Characterization of PCL-Based Electrospun Fibers

**Figure 1:**
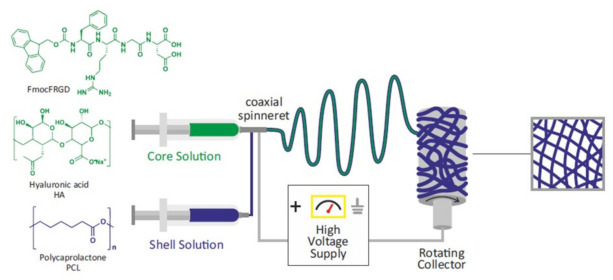
Schematic illustration of the electrospinning system used to fabricate the nanofibrous scaffolds, and the molecular structures of the different components used.

A pure PCL electrospun system was selected as the main polymer for the scaffold developed in this study, since this material has previously been used to prepare scaffolds for bone tissue regeneration [4,7,8,9]. Here, we improved the bioactivity of the scaffold by incorporating the HA polysaccharide and FmocFRGD self-assembling peptide, representing two additional bioactive components, into the elctrospun fibers. Fibrous scaffolds were designed and prepared by electrospinning nonwoven nanofiber meshes, as depicted schematically in Figure 1. Four types of meshes with different compositions were produced: (a) pure PCL fibers, (b) PCL+ FmocFRGD peptide fibers, (c) HA/PCL core/shell fibers, and (d) HA+ FmocFRGD peptide/PCL core/shell fibers. In a typical procedure, the components were dissolved in the appropriate solvents and dispensed through a single needle, to obtain single component fibers, or through a coaxial needle in the case of core/shell fibers. A high voltage was applied between the needle and grounded rotating drum collector, and the charged polymer solutions were jetted towards the collector. The jet was thinned and dried throughout the process, to form a nanofiber that was deposited on the rotating drum. In order to increase the size of the scaffold sheets and to improve the homogeneity of the scaffold, the dispensing syringe was positioned on a moving stage and was repeatedly moved back and forth, until the entire solution was spun and the required thickness was achieved.

### 2.2. Characterization and Surface Properties of the Electrospun Fibers

**Figure 2:**
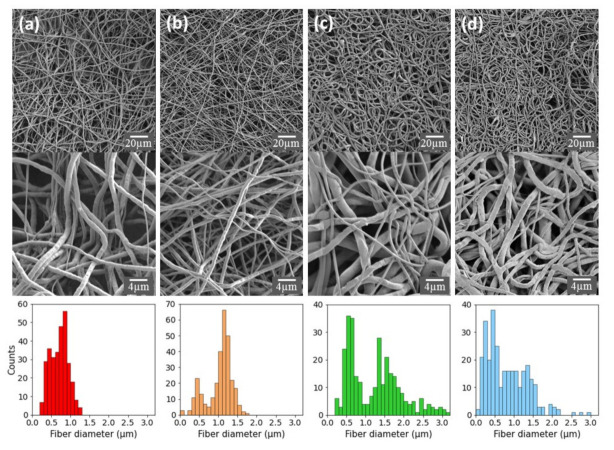
SEM micrographs (top), zoom in on the fibers (middle), and fiber diameter distribution (bottom) for electrospun meshes of (**a**) polycaprolactone (PCL), (**b**) PCL + Fmoc-phenylalanine-arginine-glycine-aspartic acid (FmocFRGD), (**c**) hyaluronic acid (HA)/PCL core/shell, and (**d**) HA + FmocFRGD/PCL core/shell fibers.

The morphology of the nonwoven fibrous meshes and the distribution of fiber diameters were analyzed and characterized by scanning electron microscopy (SEM, Figure 2). The SEM micrographs indicate that the meshes are homogeneous and exhibit a 3D porosity. While the pure PCL fibers exhibit a relatively homogeneous size distribution, with an average diameter of 0.7 ± 0.2 µm, the addition of FmocFRGD peptide to the PCL yielded a bimodal distribution with a smaller population of fibers having an average diameter of 0.5 µm and a larger population of fibers with an average diameter of 1.2 µm. A bimodal distribution was also observed for the HA/PCL core/shell fibers, with one population centered around a diameter of 0.6 µm, and another population of fibers with an average diameter of 1.5 µm that exhibits a broader diameter distribution. The HA + FmocFRGD/PCL core/shell fibers exhibit a unimodal distribution with a positive skew, with fiber diameters ranging from 100 nm to 2 µm. The various fiber distributions can be seen in the high-magnification images of the different samples (Figure 2, middle).

The fibrous mesh structure and morphology are similar to the fibrillary nature of the ECM, which is a crucial characteristic for a scaffold used in tissue engineering because of the active interaction between the cells and the ECM [4,6,33]. Cells sense the ECM and react to it under normal physiological conditions. Moreover, under pathological conditions, the ECM plays a key role in the process of tissue regeneration and repair [34]. The porous architecture demonstrated by the fibrous scaffold is another important characteristic in tissue engineering, since the interconnected pores allow cellular ingrowth and diffusion of nutrients, oxygen, and waste products across the scaffold [4,5,6,33]. A scaffold composed of electrospun PCL, HA, and with an RGD motif, ideally resembles the ECM structure with respect to fibrous protein, glycosaminoglycans, and the proteins that mediate cell attachments, such as fibronectin, respectively [4,6,30,31].

**Figure 3:**
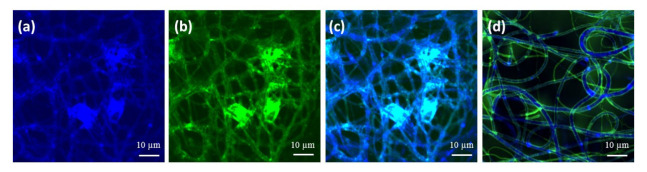
Confocal microscopy images of a dry mesh of HA + FmocFRGD/PCL core/shell fibers. (**a**) The PCL component (blue). (**b**) The HA + FmocFRGD component (green). (**c**) The overlay of the two components. (**d**) The same sample after immersion in water, displaying the transition of the HA + FmocFRGD (green) to the surface of the fibers, leaving the PCL (blue) at the center of the fibers.

To further analyze the morphology of the obtained core/shell fibers, trace amounts of polymeric fluorescent dyes were added to the core and shell solutions, and the scaffolds were imaged in a confocal microscope. Figure 3 shows the confocal images of the dry HA + FmocFRGD/PCL core/shell fibers mesh. Despite jetting the components in a core/shell configuration, the HA + FmocFRGD peptide component (Figure 3a, green) and the PCL component (Figure 3b, blue) occupy the entire volume of the fibers, and their overlay indicates that they mixed during the jetting process (Figure 3c). Upon immersing the scaffold in water (Figure 3d), the morphology of the fibers changes: The hydrophilic components (HA + FmocFRGD) extend towards the aqueous solution, and the HA and RGD groups become exposed at the surface of the fibers, while the PCL becomes the core of the fiber (Figure 3d). Supplementary Materials Figure S1 demonstrates the confocal images of HA/PCL core/shell fibers.

**Figure 4:**
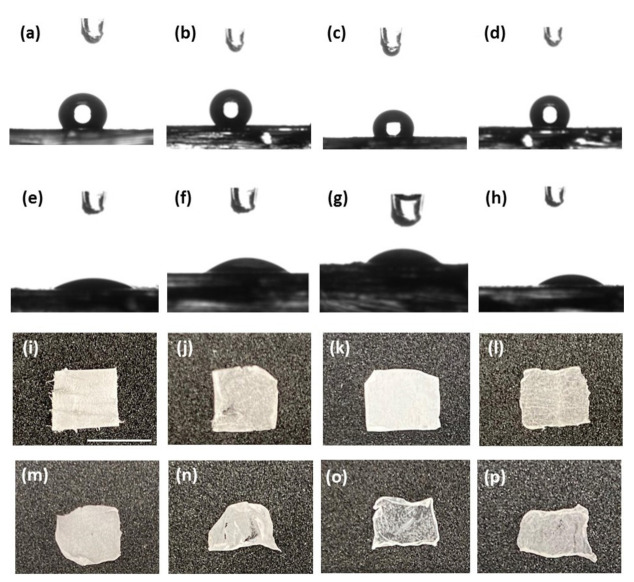
Characterization and surface properties of HA, FmocFRGD, and PCL electrospun fibers. (**a**–**d**) Images of a water droplet on (**a**) PCL, (**b**) FmocFRGD + PCL, (**c**) HA/PCL core/shell, and (**d**) HA + FmocFRGD/PCL core/shell scaffolds. (**e**–**h**) Images of a water droplet after plasma treatment on (**e**) PCL, (**f**) FmocFRGD + PCL, (**g**) HA/PCL core/shell, and (**h**) HA + FmocFRGD/PCL core/shell scaffolds. (**i**–**l**) Images of the electrospun scaffolds (**i**) PCL, (**j**) FmocFRGD + PCL, (**k**) HA/PCL core/shell, and (**l**) HA + FmocFRGD/PCL core/shell. (**m**–**p**) Images of the electrospun scaffolds after 24 h immersion in cell culture media: (**m**) PCL, (**n**) FmocFRGD + PCL, (**o**) HA/PCL core/shell, and (**p**) HA + FmocFRGD/PCL core/shell. Scale bar = 7 mm.

As the next stage, we studied the wettability of the different scaffolds, since wettability is an important characteristic for scaffolds in tissue engineering [15,33]. Contact angle measurements were performed to assess the hydrophobicity of the scaffolds (Figure 4i–l). In general, all surfaces appeared hydrophobic, probably due to the hydrophobic nature of the PCL. However, the FmocFRGD + PCL scaffold demonstrated the highest hydrophobicity (Supplementary Materials Figure S2). This may be due to the arrangement of the aromatic amino acids in the scaffold. As expected, the HA/PCL core/shell scaffold exhibits low hydrophobicity (Supplementary Materials Figure S2), due to the contribution of the hydrophilic HA. To increase the hydrophilicity, the meshes were treated with oxygen plasma, which introduces polar functional groups to the surface and is widely used to improve the hydrophilic nature of materials [15,33,35]. Oxygen plasma treatment was performed for 40 s, at a low intensity, on the scaffolds, and resulted in a significant increase in the hydrophilic characteristics (Figure 4e–h). Macroscopically, all the scaffolds appear smooth, with similar surface properties (Figure 4i–l). The fibers are visible and not ordered. Figure 4m–p presents the different scaffolds after a 24 h immersion in cell culture media. The size and shape remained well-preserved; however, when the scaffolds were removed from the medium, the edges slightly folded.

### 2.3. Biocompatibility of the HA + FmocFRGD/PCL Core/Shell Electrospun Fibers

**Figure 5:**
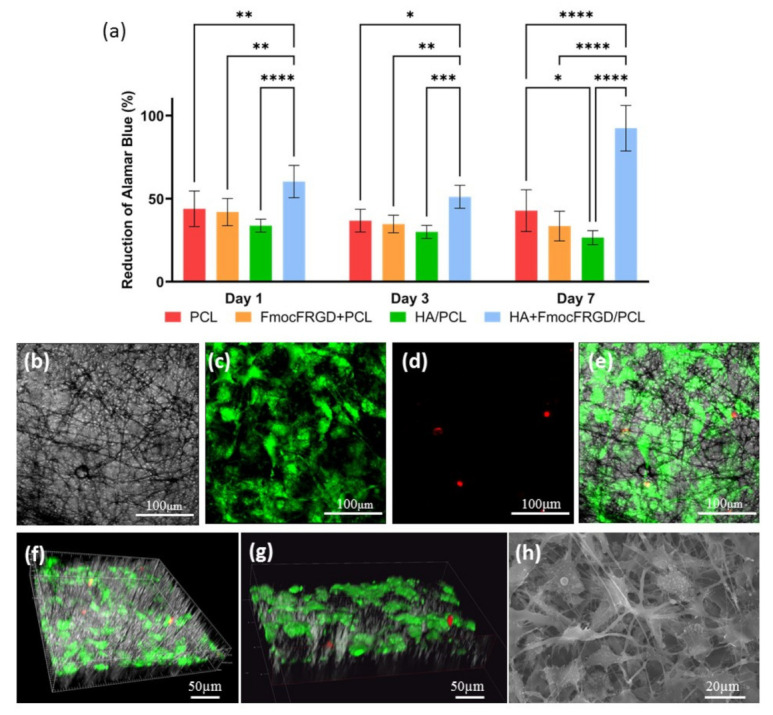
MC3T3-E1 cell viability and spreading on HA + FmocFRGD/PCL core/shell electrospun fibers. (**a**) Number of viable MC3T3-E1 cells at different time points evaluated by Alamar blue. (**b**–**e**) MC3T3-E1 cells cultured for three days on an HA + FmocFRGD/PCL core/shell scaffold and stained with fluorescein diacetate and propidium iodine. (**f**–**g**) 3D reconstruction of an HA + FmocFRGD/PCL core/shell scaffold. (**h**) SEM image of MC3T3-E1 cells on an HA + FmocFRGD/PCL core/shell scaffold. Data were analyzed by using two-way ANOVA. * *p* < 0.05, ** *p* < 0.01, *** *p* < 0.001, and **** *p* < 0.0001.

In vitro cell viability experiments were performed, to estimate the potential of the material to serve as a scaffold in tissue engineering. MC3T3-E1 preosteoblasts cells were seeded on the different scaffolds, and the cell viability was evaluated with Alamar blue, one, three, and seven days after seeding. Following seven days of incubation, the HA + FmocFRGD/PCL core/shell samples demonstrated a high biocompatibility of 92%, while the other scaffolds exhibited a biocompatibility of up to 50% (Figure 5a). Live/dead staining with fluorescein diacetate, a cell membrane dye that stains live cells green, and propidium iodine, a DNA dye that stains dead cells red, was carried out three days after seeding. The scaffolds were visualized by confocal microscopy, to examine the viability of the attached cells, their ability to spread inside the scaffold, and their shape. The results revealed that the HA + FmocFRGD/PCL core/shell scaffold were highly populated by MC3T3-E1 cells with normal morphology (Figure 5b–e). Live/dead staining was performed on the various scaffolds: FmocFRGD + PCL, HA/PCL core/shell and PCL scaffolds (Supplementary Materials Figures S3–S5). Although high numbers of cells were detected on the FmocFRGD + PCL scaffold, many of the cells were stained with propidium iodine (Supplementary Materials Figure S3). Lower numbers of cells were seen on the HA/PCL core/shell and PCL scaffolds, but while the majority of cells on the HA/PCL core/shell was viable, some of the cells on the PCL scaffold were dead (Supplementary Materials Figures S4–S5). Moreover, 3D visualization of the HA + FmocFRGD/PCL scaffolds revealed a homogenous distribution of the cells on the scaffold, and their attachment to the fibers (Figure 5f–g). Figure 5h presents the cellular adhesion to the fibers, as visualized by SEM. These observations highlight the contribution of HA and FmocFRGD peptide to the biocompatibility, and cell-attachment properties of the scaffold.

### 2.4. Osteogenesis of HA + FmocFRGD/PCL Core/Shell Electrospun Fibers

**Figure 6:**
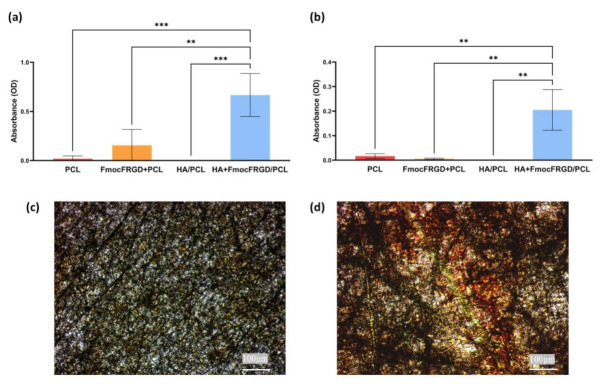
Osteogenesis on HA + FmocFRGD/PCL core/shell electrospun fibers. (**a**) Quantification of Alkaline Phosphatase (ALP) activity of MC3T3-E1 cells, 16 days after seeding with osteogenic differentiation media. (**b**) Quantification of calcification by Alizarin red (AR) staining, 16 days after seeding in osteogenic differentiation media. (**c**, **d**) Optic microscope images of MC3T3-E1 preosteoblast cells stained with Alizarin red. (**c**) MC3T3-E1 cells 16 days after seeding on PCL fibers. (**d**) MC3T3-E1 cells 16 days after seeding on HA + FmocFRGD/PCL core/shell fibers. Data were analyzed by using a one-way ANOVA. * *p* < 0.05, ** *p* < 0.01 and *** *p* < 0.001.

Cell differentiation was assessed, to examine the capability of HA + FmocFRGD/PCL core/shell fibers to induce differentiation of MC3T3-E1 preosteoblast cells into mature osteoblasts cells. ALP activity is crucial for bone mineralization; hence, ALP is a useful biochemical marker for bone formation [36,37]. MC3T3-E1 preosteoblast cells were seeded on the various fibrous scaffolds and cultured for 16 days. After this time, the ALP substrate, 4-methylumbelliferyl phosphate (4-MUP), was added to the cells, to evaluate the intracellular ALP activity. Figure 6a shows higher ALP activity in the differentiated cells seeded on the HA + FmocFRGD/PCL core/shell fibers, compared to cells seeded on scaffolds without HA or FmocFRGD peptide, indicating the osteogenic potential of including HA FmocFRGD. Mature osteoblast cells secrete extracellular calcium during mineralization, which can be identified by Alizarin red staining [38]. Figure 6b demonstrates the increased intensity of the red stain in the differentiated cells growing on HA + FmocFRGD/PCL core/shell fibers, in comparison to the scaffolds without HA or FmocFRGD peptide. These results demonstrate the effect of HA and FmocFRGD on cellular differentiation and the ability of these fibers to support biomineralization. Figure 6c,d presents the mineral bone nodule formation, as seen by Alizarin red staining in the differentiated cells on the HA + FmocFRGD/PCL core/shell fibers, in comparison to the low bio-mineralization on the PCL scaffold. Taken together, the viability and differentiation of MC3T3-E1 preosteoblasts on the HA + FmocFRGD/PCL core/shell scaffolds, as evidenced by high ALP activity and Alizarin red staining, confirm the important contribution of the combination of HA and FmocFRGD peptide, and demonstrate the potential of these scaffolds for use in bone tissue regeneration.

## 3. Materials and Methods

### 3.1. Materials

Lyophilized FmocFRGD was purchased from GL Biochem (Shanghai, China). High-molecular-weight hyaluronic acid (3 × 10^6^ Da) was purchased from BTG-Ferring, Kiryat Malachy, Israel. Alamar blue was purchased from Enco (Petah Tikva, Israel). Minimum Essential Medium Eagle (α-MEM) was purchased from Rhenium (Modi’in-Maccabim-Re’ut, Israel). Fetal calf serum, penicillin, and streptomycin were purchased from Biological Industries Beit Haemek (Beit Haemek, Israel). Polycaprolactone (average Mn 80,000) Fluorescein 5(6)-isothiocyanate (FITC), Fluorescein diacetate, poly (2,5-dihexyloxy-1,4-phenylenevinylene), propidium iodide, Alizarin red, and 4-Methylumbelliferyl phosphate (4-MUP) were purchased from Sigma-Aldrich (Rehovot, Israel). MC3T3-E1 preosteoblast cells were purchased from ATCC (Manassas, VA, USA).

### 3.2. Methods

#### 3.2.1. Preparation of the Scaffolds

The electrospinning setup contained two syringe pumps (New Era Pump Systems, Inc., Farmingdale, NY, USA), a power supply (DC voltage source, Gamma High Voltage Research, Ormond Beach, FL 32174, USA), a moving stage, and a rotating drum collector. In a typical procedure, a polymer solution is dispensed through a syringe at a controlled rate dictated by the syringe pump, while applying a high voltage between the syringe needle and a conductive collector.

Non-woven meshes were formed by positioning the dispensing syringe on a moving stage and repeatedly moving the syringes back and forth at a velocity of 0.1 cm/s, until the entire solution was spun and a nonwoven mesh with a thickness of 40 µm was attained. The fibers were collected on a grounded rotating drum at a tip-to-ground distance of 7 cm and at a drum rotation speed of 80 rpm. The total area of the electrospun meshes were 13 cm × 30 cm (390 cm^2^). The electrospinning was performed at ambient conditions (average temperature of 22 °C and average relative humidity of 60%).

For the electrospinning of PCL fibers, 0.225 g of PCL was dissolved in 1.5 mL of hexafluoroisopropanol (HFIP) (15% (*w*/*v*)), to obtain a clear homogeneous solution. The solution was dispensed at a rate of 0.450 mL/h, through a syringe with a 25-gauge needle, under a driving voltage of 7 kV.

For the electrospinning of PCL FmocFRGD fibers, FmocFRGD was first dissolved in HFIP, at a concentration of 50 mg/mL. Next, the stock solution was added to the solution of the PCL in HFIP, described above. The solutions were mixed to form a final composition of 15% (*w*/*v*) of PCL and 0.125% (*w*/*v*) of FmocFRGD. Fresh stock solution was prepared for each experiment, and the final combined solution was used immediately. The 1.5 mL solution was dispensed at a rate of 0.450 mL/h, through a syringe with a 25-gauge needle, under a driving voltage of 7 kV.

For the electrospinning of HA/PCL core/shell fibers, commercial HA (1% (*w*/*v*)) was diluted with distilled water, to provide a 1.0 mL solution with a concentration of 0.5% (*w*/*v*), while the PCL solution was prepared by dissolving 0.400 g of PCL in 2.0 mL of HFIP (20% (*w*/*v*)). The two solutions were dispensed simultaneously through a coaxial needle composed of a 25-gauge core needle and a 14-gauge shell needle. HA was dispensed at a rate of 0.450 mL/h, while PCL was dispensed at a rate of 0.900 mL/h, under a driving voltage of 19 kV.

For electrospinning HA + FmocFRGD/PCL core/shell fibers, the FmocFRGD was first dissolved in DMSO, at a concentration of 100 mg/mL. Next, the FmocFRGD stock solution in DMSO was mixed with the diluted HA solution in distilled water, to give a 1.0 mL solution of 0.125% (*w*/*v*) FmocFRGD and 0.5% (*w*/*v*) HA in 98.75:1.25 v/v water:DMSO mixture. The mixture was vortexed until fully mixed. Fresh stock solution was prepared for each experiment, and the final combined solution was used immediately. The PCL solution was prepared by dissolving 0.400 g of PCL in 2.0 mL of HFIP (20% (*w*/*v*)). The two solutions were dispensed simultaneously through a coaxial needle composed of a 25-gauge core needle and a 14-gauge shell needle of. the HA was dispensed at a rate of 0.450 mL/h, while the PCL was dispensed at a rate of 0.900 mL/h, under a driving voltage of 17 kV.

#### 3.2.2. High-Resolution Scanning Electron Microscopy

High-resolution scanning electron microscopy was performed in a Zeiss Gemini 300 field-emission scanning electron microscope (FESEM), in a high vacuum mode, at a work distance (WD) ≈ 5 mm and voltage of 1–3 kV, without coating of the samples. The size distributions of the fibers were obtained from the SEM micrographs by measuring the diameters of ~200 fibers from each sample, using the ImageJ Image processing software [39].

#### 3.2.3. Water Contact Angle Measurements

The water contact angle of the fiber meshes was measured, using the Ramé-Hart goniometer with the Drop Image Advance analysis software, at room temperature. A drop of DDW (2 µL) was deposited on the scaffolds, and the average contact angle was calculated from 20 measurements on each surface.

#### 3.2.4. Cell Viability Assay

Murine MC3T3-E1 preosteoblast cells were cultured in α-MEM supplemented with bovine serum and 100 UmL^−1^ penicillin and 100 UmL^−1^ streptomycin, in a Petri dish, and incubated at 37 °C, in a humidified atmosphere containing 5% CO_2_. The fibrous scaffolds were cut into 7 × 7 mm^2^, placed I sn 96-well cell repellent plates, and sterilized by UV for 30 min. Cells were seeded on the fibers at a density of 5 × 10³ cells/well and left at 37 °C, in a humidified atmosphere containing 5% CO_2_. Cell viability was assessed by using the Alamar blue assay at 1, 3, and 7 days after seeding. The medium was removed from the wells at each time point, and a solution of 10 µL of Alamar blue and 90 µL of α-MEM medium was added to each well, followed by 4 h of incubation. After the incubation period, absorbance of each well was measured, using a Tecan Spark plate reader at 570 nm wavelength (reduced) and 600 nm (oxidized). The percentage of reduced Alamar blue was calculated from the equation provided by the materials manufacturer.

#### 3.2.5. Live/Dead Assay

Qualitative assessment of cell viability on the scaffold was performed by using the live/dead staining kit. Fibrous scaffolds were cut into 7 × 7 mm^2^, put in 96-well plates, and UV sterilized for 30 min. MC3T3-E1 cells were then seeded on the fibers, at a density of 1.2 × 10^4^ cells/well, and were incubated for 72 h. Live/dead staining solution containing fluorescein diacetate (6.6 µg/mL) and propidium iodide (5 µg/mL) was used to label the live and dead cells in green and red, respectively. The medium was removed from the wells, and 50 µL of the staining solution was added to each well. The cells were immediately imaged, using a Leica SP8 X Confocal Microscope.

#### 3.2.6. Alkaline Phosphatase (ALP) Activity

To determine the intracellular ALP activity, the fibrous scaffolds were cut into 7 × 7 mm^2^ and placed in 96-well plates, followed by 30 min of UV sterilization. MC3T3-E1 preosteoblasts cells were seeded at a density of 5 × 10^3^ cells/well. After 2 days of incubation, the medium was replaced with differentiation medium, which was changed every 2 days, for a period of 14 days. After 14 days, the fibers were treated with 100 µL of ALP substrate solution containing 4-Methylumbelliferyl phosphate (4-MUP) and incubated for 30 min, in the dark. Finally, absorbance was measured at 405 nm.

#### 3.2.7. Alizarin Red Staining

Alizarin red staining assays were performed in order to evaluate the extent of matrix calcification. The fibrous scaffolds were cut into 7 × 7 mm^2^ and placed in 96-well plates, followed by 30 min UV sterilization. MC3T3-E1 preosteoblasts cells were seeded at a density of 5 × 10^3^ cells/well. After 2 days of incubation, the medium was replaced with differentiation medium, which was changed every 2 days, for a period of 14 days. After 14 days, cells were washed with PBS and fixed for 30 min with 70% ethanol. Following cell fixation, cells were exposed to Alizarin red solution for 30 min. After washing off the excess dye, optical light images were taken from each well, using a Nikon Eclipse Ts2 Microscope. The Alizarin red stain was dissolved in a solution of methanol and acetic acid (2:1), and the absorbance was measured at 405 nm, to quantitate the result.

#### 3.2.8. Statistical Analysis

Statistical analysis was performed by using GraphPad Prism Software (GraphPad Software, Inc., San Diego, CA, USA). Data for each assay were analyzed with one-way analysis of variance (ANOVA) or two-way ANOVA. Statistical significance was set at α = 0.05.

## 4. Conclusions

In this paper, we described a scaffold designed for bone tissue engineering applications, made of electrospun fibers composed of PCL, together with an FmocFRGD peptide incorporated into HA. The objective was to add the favorable biocompatibility and cellular attachment properties of HA and the RGD moiety, respectively, to a PCL-based scaffold. The resultant fibrous scaffold morphologically resembles the fibrillar structure of the ECM and exhibits a high degree of biocompatibility. In addition, the fibrous scaffold facilitated osteogenic differentiation, as evidenced by intracellular ALP activity and biomineralization. These results suggest that the HA + FmocFRGD/PCL core/shell composite scaffold has potential use in bone tissue regeneration.

## Data Availability

Not applicable.

## Supplementary Materials

The following are available online, at https://www.mdpi.com/1422-0067/22/5/2425/s1. Figure S1: Confocal micrograph of the HA/PCL core/shell fibers scaffold. (a) The PCL component, (b) the HA component, and (c) overlay of the components. Figure S2: Live/dead assay of MC3T3-E1 cells on FmocFRGD + PCL fibers. MC3T3-E1 cells cultured for three days and stained with fluorescein diacetate and propidium iodine: (a) brightfield image, (b) fluorescein diacetate stain, (c) propidium iodine stain, and (d) merged image. Figure S3: Live/dead assay of MC3T3-E1 cells on HA/PCL core/shell fibers. MC3T3-E1 cells cultured for three days and stained with fluorescein diacetate and propidium iodine: (a) brightfield image, (b) fluorescein diacetate stain, (c) propidium iodine stain, and (d) merged image. Figure S4: Live/dead assay of MC3T3-E1 cells on PCL fibers. MC3T3-E1 cells cultured for three days and stained with fluorescein diacetate and propidium iodine: (a) brightfield image, (b) fluorescein diacetate stain, (c) propidium iodine stain, and (d) merged image. Figure S5: Water contact angle measurements of PCL, FmocFRGD + PCL, HA/PCL core/shell, and HA + FmocFRGD/PCL core/shell scaffolds before plasma treatment.
