# Supplementary material for: Hyaluronic Acid and a Short Peptide Improve the Performance of a PCL Electrospun Fibrous Scaffold Designed for Bone Tissue Engineering Applications
Source: Int J Mol Sci. 2021 Mar 12;22(5):2425. doi: 10.3390/ijms22052425 (PMC7975971; doi:10.3390/ijms22052425)
Supplement: Supplementary file 1 [file ijms-22-02425-s001.pdf]

Supplementary Materials

Hyaluronic Acid and a Short Peptide Improve the Performance of a PCL Electrospun Fibrous Scaffold Designed for Bone Tissue Engineering Applications

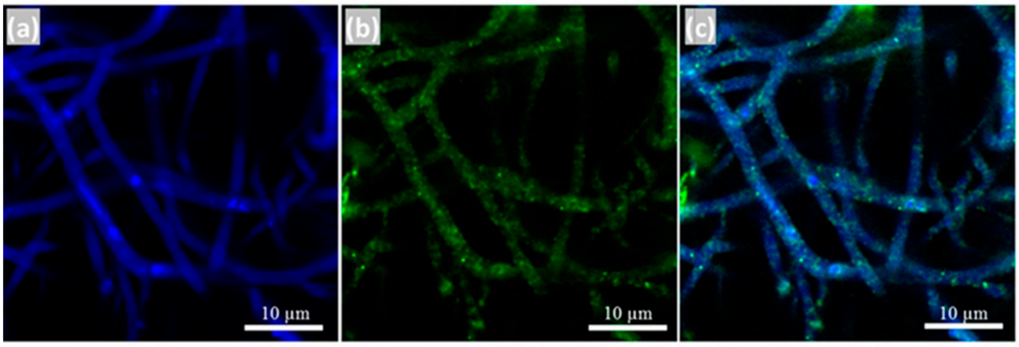

Figure S1. Confocal micrograph of the HA/PCL core/shell fibers scaffold. (**a**) The PCL component, (**b**) the HA component, and (**c**) The overlay of the two components.

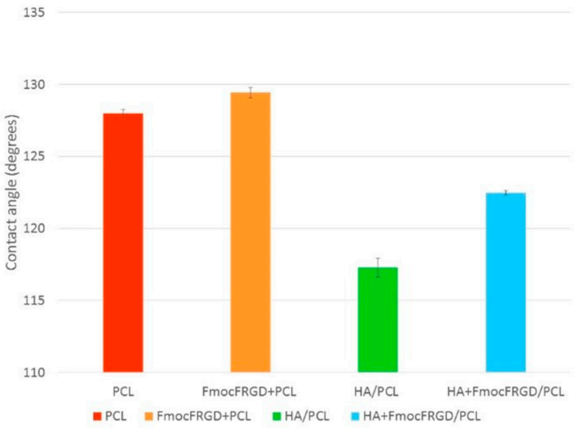

Figure S2. Water contact angle measurements of PCL, FmocFRGD+PCL, HA/PCL core/shell and HA+FmocFRGD/PCL core/shell scaffolds before plasma treatment.

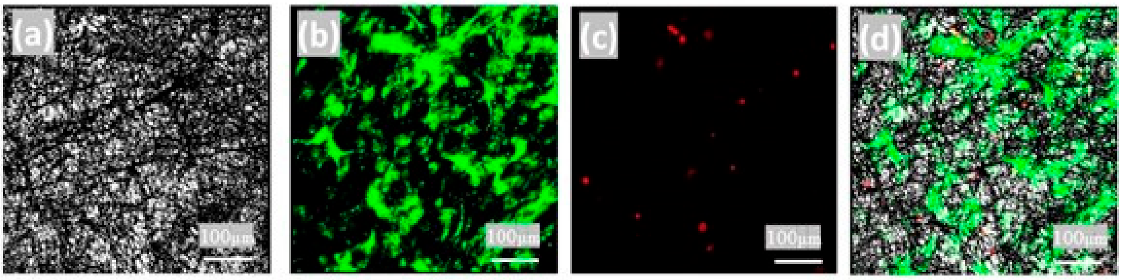

Figure S3. Live/Dead assay of MC3T3-E1 cells on FmocFRGD+PCL fibers. MC3T3-E1 cells cultured for 3 days and stained with fluorescein diacetate and propidium iodine. (**a**) brightfield image, (**b**) fluorescein diacetate stain, (**c**) propidium iodine stain, (**d**) merged image.

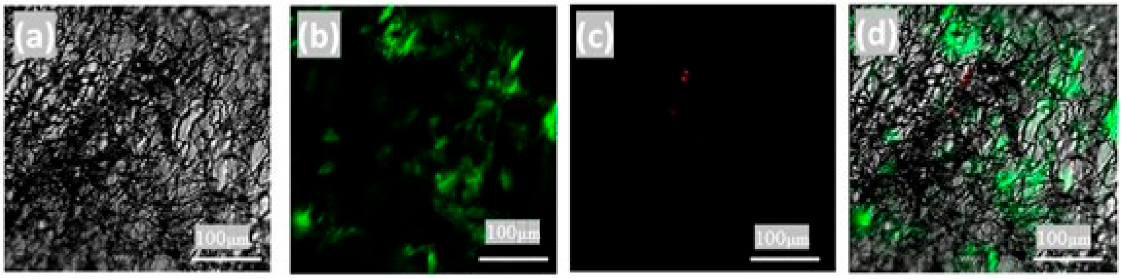

Figure S4. Live/Dead assay of MC3T3-E1 cells on HA/PCL core/shell fibers. MC3T3-E1 cells cultured for 3 days and stained with fluorescein diacetate and propidium iodine. (**a**) brightfield image, (**b**) fluorescein diacetate stain, (**c**) propidium iodine stain, (**d**) merged image.

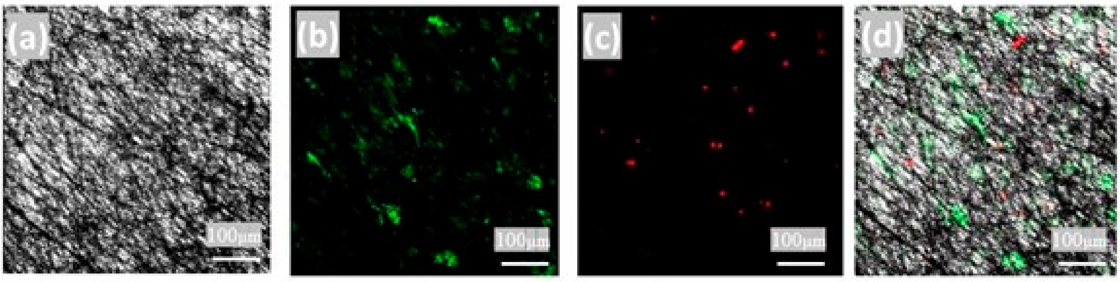

Figure S5. Live/Dead assay of MC3T3-E1 cells on PCL fibers. MC3T3-E1 cells cultured for 3 days and stained with fluorescein diacetate and propidium iodine. (**a**) brightfield image, (**b**) fluorescein diacetate stain, (**c**) propidium iodine stain, (**d**) merged image.
